# Interventions to improve daily medication use among adolescents and young adults: what can we learn for youth pre-exposure prophylaxis services?

**DOI:** 10.1097/QAD.0000000000002777

**Published:** 2020-12-14

**Authors:** Jennifer Velloza, Bill Kapogiannis, Linda-Gail Bekker, Connie Celum, Sybil Hosek, Sinead Delany-Moretlwe, Rachel Baggaley, Shona Dalal

**Affiliations:** a Department of Global Health, University of Washington, Seattle, Washington; b Maternal and Pediatric Infectious Disease Branch, Eunice Kennedy Shriver National Institute of Child Health and Human Development, Bethesda, Maryland, USA; c The Desmond Tutu HIV Centre, University of Cape Town, Cape Town, South Africa; d Department of Medicine, University of Washington, Seattle, Washington; e Department of Psychiatry, Stroger Hospital of Cook County, Chicago, Illinois, USA; f Wits Reproductive Health and HIV Institute (Wits RHI), Faculty of Health Sciences, University of the Witwatersrand, Johannesburg, South Africa; g Global HIV, Hepatitis and STIs Programmes, World Health Organization, Geneva, Switzerland.

**Keywords:** adherence, adolescents and young adults, antiretroviral therapy, asthma, diabetes, oral contraceptives, pre-exposure prophylaxis

## Abstract

**Objective:**

Oral pre-exposure prophylaxis (PrEP) is an important HIV prevention method and studies have shown that young people ages 15–24 have difficulty adhering to daily PrEP. The field of PrEP delivery for young people is relatively nascent and lessons about potential PrEP adherence interventions could be learned from the larger evidence base of adherence interventions for other daily medications among youth.

**Design:**

Systematic review of adherence support interventions for adolescents.

**Methods:**

We searched PubMed, CINAHL, EMBASE, and PsycINFO through January 2020 for oral contraceptive pill (OCP), antiretroviral therapy (ART), asthma, and diabetes medication adherence interventions. We reviewed primary articles about OCP adherence interventions and reviewed systematic reviews for ART, asthma, and diabetes medication adherence interventions. Studies were retained if they included participants’ ages 10–24 years; measured OCP, ART, asthma, or diabetes medication adherence; and were systematic reviews, randomized trials, or quasi-experimental studies.

**Results:**

Fifteen OCP articles and 26 ART, diabetes, and asthma systematic reviews were included. Interventions that improved medication adherence for OCPs, ART, asthma, and diabetes treatment included reminder text messages, computer-based and phone-based support, and enhanced counseling. Multi-month prescriptions and same-day pill starts also were found to improve OCP adherence and continuation. Adolescent-friendly clinics and peer-based counseling significantly improved ART adherence, and telemedicine interventions improved diabetes medication adherence.

**Conclusion:**

Interventions that improve medication adherence among youth include enhanced counseling, extended pill supply, adolescent-friendly services, and text message reminders. PrEP programs could incorporate and evaluate such interventions for their impact on PrEP adherence and continuation among at-risk adolescents.

## Introduction

Adolescent girls and young women are at high risk of HIV in high-burden countries, as are young key populations in all regions and may substantially benefit from pre-exposure prophylaxis (PrEP) [1–4]. However, efficacy trials and demonstration projects have shown that youth have difficulty adhering to and persisting with daily oral PrEP [5–10]. For example, in the Adolescent Medicine Trials Network for HIV/AIDS Interventions (ATN) 110 and 113 studies and the PrEP Brasil demonstration study, young MSM had declining PrEP adherence over time [10–12]. The PlusPills Study with adolescent girls in Cape Town found similar adherence drops offs when clinic visits moved from monthly to quarterly [6].

PrEP programs are now focused on identifying PrEP adherence approaches to maximize its public health impact [13,14]. Behavior change theories postulate that adherence could be improved by influencing information about medication dosing and side effects, increasing motivation and social support around taking medication, improving skills and self-efficacy to use medication, and increasing availability and access [15,16]. Based on these models, PrEP projects have used a combination of adherence support targeting information, motivation, support, skills, and self-efficacy, including counseling, two-way text message communication with providers and text reminders, drug-level feedback, adherence support clubs, integrated PrEP delivery with sexual and reproductive health, and adolescent-friendly clinics [6,17–20]. While these approaches have been designed to address PrEP adherence challenges for young people, they are typically tested together as a package, making it difficult to estimate the impact of each approach alone compared with standard-of-care services [13]. Gaps remain in understanding the strengths, limitations, and effectiveness of various adherence support approaches currently under evaluation in PrEP trials and identifying other support strategies to address adolescent concerns around PrEP use.

PrEP implementation is scaling up, but challenges remain for adolescents. There is a larger evidence base from other prevention and disease management interventions for adherence among youth such as oral contraceptive pills (OCPs), antiretroviral therapy (ART), and daily medications for chronic disease management (e.g. asthma, diabetes). Interventions in these areas target theoretical mediators of daily medication use that may be relevant for improving PrEP adherence and continuation. While specific motivators of PrEP adherence among youth may differ from those driving prevention of pregnancy or chronic disease progression, consistencies across this literature can provide insights on next directions for PrEP interventions. We conducted a systematic review on intervention strategies for daily OCP, ART, asthma, and diabetes medication use among adolescents and young adults to identify lessons learned about adherence support approaches that could have relevance for PrEP programs for youth. OCPs are likely most similar to PrEP because they are also recommended for daily use, only during periods of need, in an otherwise healthy population, and we pay particular attention to primary articles from the OCP field. We also reviewed systematic reviews for ART, asthma, and diabetes medication interventions as these also require daily adherence over a long period of time.

## Methods

We conducted a review of interventions (e.g. randomized trials, quasiexperimental studies) that assessed OCP uptake, adherence, and continuation; and a review of systematic reviews focused on ART, diabetes, and asthma medication adherence among youth. We chose to conduct a review of systematic reviews for the second component, rather than a review of original research, because a number of relevant reviews have already been done. This work was conducted in line with the Preferred Reporting Items for Systematic Reviews and Meta-Analyses guidelines [21].

### Search strategy

**Fig. 1:**
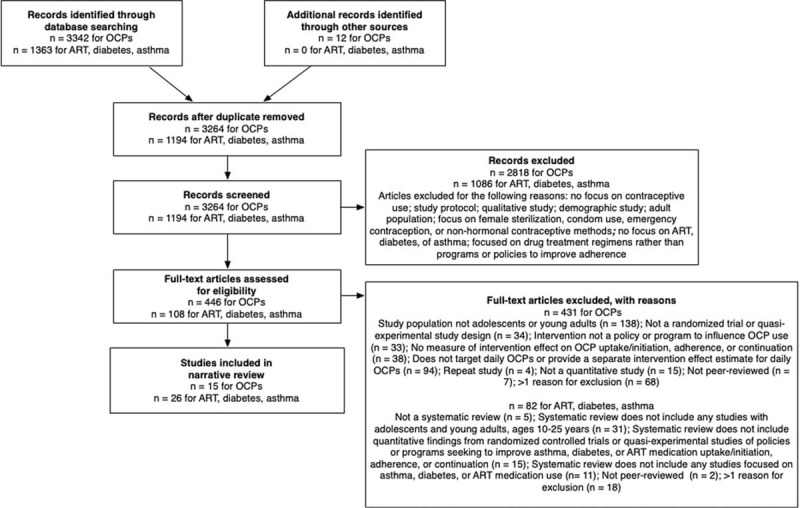
Flowchart of reviewed and included studies.

We searched PubMed, CINAHL, PsycINFO, and EMBASE for primary articles on OCP interventions and systematic reviews focused on ART, diabetes, and asthma medication among youth, published through 24 January 2020 (refer to Fig. 1 footnote for search terms).

Search terms for oral contraceptive pill articles: our search included terms for contraceptive use (e.g. ‘contraceptive agents’, ‘birth control’, ‘family planning’), adolescents and young adults (e.g. ‘adolescent’, ‘youth’, ‘girl’, ‘teen’), intervention studies (e.g. ‘randomized controlled trial’, ‘comparative study’, ‘follow-up study’), and medication use (e.g. ‘medication adherence’, ‘medication compliance’, ‘continuation’, ‘adoption’, ‘contraceptive device utilization’). Search terms for antiretroviral therapy, diabetes, and asthma articles: we searched databases with terms to identify antiretroviral therapy use (e.g. ‘ART’, ‘antiretroviral’, ‘anti-HIV agents’), diabetes and asthma medication (e.g. ‘chronic disease drug therapy’, ‘diabetes’, ‘diabetes mellitus drug therapy’, ‘oral hypoglycemic agents’, ‘asthma’, ‘anti-asthmatic agents’, ‘asthma drug therapy’), adolescents and young adults (e.g. ‘adolescent’, ‘youth’, ‘teen’), review studies (e.g. ‘systematic review’, ‘meta-analysis’), and medication use (e.g. ‘medication adherence’, ‘medication compliance’, ‘continuation’, ‘adoption’). The numbers provided for antiretroviral therapy, diabetes, and asthma articles reflect the number of systematic reviews included/excluded rather than the number of individual studies from these reviews. ART, antiretroviral therapy; OCP, oral contraceptive pills.

### Study selection

OCP use studies were included if they met the following inclusion criteria: first, population included female adolescents or young adults (ages 10–24 years) and there was a separate effect measure for adolescents or young adults; second, study was a randomized trial or quasi-experimental study; third, contraceptive adherence or continuation were included as outcomes; and fourth, interventions focused on daily OCP use and were intended to influence contraceptive use.

ART, diabetes, and asthma medication reviews were included if they included at least one study with youth ages 10–24 years; were systematic reviews; and included interventions for ART, diabetes, or asthma medication use.

Two reviewers (J.V. and S.D.) assessed 10% of full-text articles for inclusion; discrepancies were resolved through discussion. One reviewer (J.V.) completed the full-text review of the remaining articles.

### Data extraction and synthesis

Reviews on ART, diabetes, and asthma generally included a mix of adult-focused and adolescent focused-articles. We included data from the studies that included adolescents 10–24 years old by extracting data from the systematic review text and tables on study details and by consulting the primary articles to obtain additional details. We extracted pooled effect measures where available for interventions targeting 10–24 years old but, given that few reviews included a meta-analysis with adolescent trials, we primarily focused on extracting effect measures from individual studies.

## Results

### Study characteristics

We identified 3264 unique abstracts related to OCP adherence and 1194 related to studies of ART, diabetes, and asthma medication use (Fig. 1). Forty-one articles met inclusion criteria. Included studies were conducted between 1984 and 2019. Ten OCP studies were randomized trials, two were cluster randomized trials, and three were quasi-experimental studies. Sample sizes ranged from 33 to 10 600. Three studies included male participants who were asked about their partners’ OCP use. Primary studies on ART, asthma, and diabetes identified from systematic reviews were all randomized trials or quasi-experimental studies. The majority of OCP-focused studies were conducted in the United States, and one study each was conducted in China, Iceland, and Cameroon. ART, asthma, and diabetes studies were predominantly conducted in the United States, but several systematic reviews also included studies conducted in Africa, Asia, and Europe.

### Summary of interventions

Based on descriptions of the interventions provided in included articles, we grouped interventions into four categories: individual-level, interpersonal-level, health facility-level, and community-level (Table 1). For OCP-focused studies, individual-level interventions included education about OCPs, side effects, and plans for pill management; enhanced counseling using motivational interviewing or problem-solving techniques to cover topics such as contraceptive goal-setting and safe sex behaviors; and text message or phone call reminders about clinic appointments and regular pill-taking.

**Table 1:** Summary of medication adherence interventions and their efficacies among 10–24 years old, by condition.

	OCP	ART	Asthma	Diabetes
Individual level	Education and counseling^∗^Phone reminders and support^∗^	Education and counseling^∗^Computer-based counseling^∗^Phone reminders and support^∗^Financial incentivesDOT	Education and counseling^∗^Computer-based counseling^∗^Phone reminders and support^∗^DOT	Education and counseling^∗^Computer-based counseling^∗^Phone reminders and support^∗^
Interpersonal level	Peer supportPeer modeling	Family-based counseling^∗^Peer support^∗^Peel modeling^∗^	Family-based counseling^∗^Peer supportPeer modeling	Family-based counseling^∗^Peer supportPeer modeling
Health facility level	Youth-friendly clinicsSpecially trained counselorsSame-day pill starts^∗^Multi-month dispensing^∗^	Youth-friendly clinics^∗^	–	Youth-friendly clinicsTransition coordinatorsTelecare^∗^
Community level	School-based services^∗^Broad education and social marketing campaignsDecentralized OCP provision^∗^	–	School-based services multisystemic therapy^∗^	School-based services multisystemic therapy^∗^Broad education campaigns

ART, antiretroviral therapy; DOT, directly observed therapy; OCP, oral contraceptive pills.

∗ Significant intervention effects, as defined by a *P* value for the main intervention effect of less than 0.05. For ART, asthma, and diabetes interventions, this *P* value and determination of significance was made from the individual, primary articles rather than from the systematic review or any meta-analysis.

Interpersonal-level interventions included peer modeling (e.g. interacting with peers to observe them take their medications) and support groups. Health-facility level interventions focused on same-day pill starts where medications were dispensed at the visit with directly observed pill taking, regardless of menstrual cycle; multi-month pill dispensing; and youth-friendly OCP clinics and specially-trained counselors. Community-level interventions included enhanced sexual and reproductive health curriculums in schools, social marketing campaigns (e.g. OCP-related commercials and radio advertisements), and decentralized OCP provision (e.g. delivery in schools).

Interventions for ART, asthma, and diabetes were similar including counseling about adherence, phone reminders, and directly observed therapy, but also included financial incentives for ART adherence, multisystemic therapy (a community-level intervention whereby therapists involve patients and their family, teachers, and providers to address determinants of medication adherence) and family-based counseling, and transition coordinators to support the change from pediatric to adult care (Table 1).

### Effects of interventions on oral contraceptive pill use

Fifteen studies measured either OCP use (*N* = 7) or continuation (*N* = 6) following an intervention (Table 2). ‘OCP use’ was defined as current OCP use at a follow-up visit (assessed via self-report), without incorporating metrics of OCP adherence, missed pills, or missed visits. ‘OCP continuation’ was defined as continuous OCP use throughout follow-up. Interventions with statistically significant effects were those using daily text message reminders [odds ratio (OR): 1.4; 95% confidence interval (CI): 1.0–2.0 [22]], extended multi-month prescriptions (OR: 7.6; 95% CI: 2.4–24.0 [23]), same-day pill starts (OR: 1.8; 95% CI: 1.1–3.3 [21]; OR: 1.5; 95% CI: 1.0–2.1 [25]), and enhanced counseling (relative risk: 3.25; 95% CI: 1.83–5.77 [26]; *P* value = 0.05 [27]). The effect sizes ranged from ORs of 1.1 to 7.6; the highest was from a small study of 43 girls [23].

**Table 2:** Studies on improving oral contraceptive pills medication adherence among adolescents and young adults, by intervention (*N* = 15).

Year	Author	Sample size, sex, ages	Location	Study design	Intervention description	Control description	Primary outcome	Effects^a^
Enhanced clinic-based or school-based counseling								
2015	Minguez [26]	*N* = 2076 high school students; 908 women and 1168 men; 14–18 years old	USA	QS	Enhanced school-based services and decentralized OCP dispensing	SOC health services	Self-reported OCP use at 12 months	9th Graders: RR = 0.15 (95% CI: 0.02–1.46)**10–12th Graders: RR** **=** **3.25 (95% CI: 1.83–5.77)**
2012	Berenson [29]	*N* = 1155 women, 16–24 years old	USA	RCT	Enhanced clinic-based counselingEnhanced clinic-based counseling and weekly phone calls	SOC counseling	OCP continuation at 12 months, measured via self-report, pill counts, and medical records	OR = 0.80 (95% CI: 0.63–1.40)OR = 1.09 (95% CI: 0.86–1.53)
2005	Wang [33]	*N* = 2227 women, 15–24 years	China	QS	Peer-based counseling sessions	SOC counseling	Self-reported OCP use at 20 months	OR = 0.75 (95% CI: 0.52–1.08)
2004	Gilliam [31]	*N* = 33 postpartum women, <25 years old	USA	RCT	SOC counseling and post-delivery OCP counseling	SOC counseling	Self-reported OCP continuation at 12 months	OR = 0.67 (95% CI: 0.11–3.99)
2004	Bender [93]	*N* = 160 women, <24 years	Iceland	RCT	Enhanced OCP counseling	SOC counseling	Self-reported OCP use at 6 months	OR = 1.08 (95% CI: 0.54–2.14)
1997	Kirby [28]	*N* = 354 7th grade students, 12–13 years old (sex breakdown not specified)	USA	CRCT	Enhanced school-based OCP counseling	SOC curriculum	Self-reported OCP use at last sex	**OR** **=** **0.57 (95% CI: 0.36–0.91)**
1997	Kirby [34]	*N* = 10 600 middle school students, mean age 12.5 years old (sex breakdown and age range not specified)	USA	CRCT	Adult-led enhanced school-based OCP counselingPeer-led enhanced school-based OCP counseling	SOC curriculum	Self-reported OCP use at last sex	RR = 0.79 (95% CI: 0.46–1.35)RR = 0.78 (95% CI: 0.47–1.28)
1993	Hanna [27]	*N* = 51 women, 16–18 years old	USA	RCT	Enhanced clinic-based counseling	SOC counseling	Self-reported mean OCP adherence score at 3 months	**10.24 in intervention versus 8.95 in control, ratio of 1.14, *P*** **=** **0.05**
1984	Jay [32]	*N* = 57 women, 15–19 years old	USA	RCT	Peer-based counseling	SOC nurse-led counseling	Mean OCP adherence score, measured as noncompliance at 4 months measured via urinary marker for OCP use	0.85 in intervention versus 1.06 in control; ratio of 0.80
Phone and web-based interventions								
2012	Castaño [22]	*N* = 962 women, 13–25 years old	USA	RCT	Daily one-way and two-way text messages	SOC counseling	Self-reported OCP continuation at 6 months	**OR** **=** **1.44 (95% CI: 1.03–2.00)**
1999	Chewning [30]	*N* = 949 women, <20 years old, mean age 17 (age range not specified)	USA	RCT	Enhanced OCP counseling via web-based application	SOC counseling	Self-reported OCP use	Chicago: 96.6% in intervention versus 91.2% in controlMadison: 98.8% in intervention versus 97.7% in control
							Self-reported mean number of months with OCP use	Chicago: 8.2 months in intervention versus 8.6 months in controlMadison: 9.4 months in intervention versus 9.5 months in control
Multi-month dispensing								
2011	White [23]	*N* = 85 women, <18 years old (age range not specified)	USA	RCT	Increased number of pill packs (7 packs)	Standard 3 pill packs	Self-reported OCP continuation at 6 months	**OR** **=** **7.55 (95% CI: 2.38–23.95)**
Same-day pill starts								
2008	Edwards [24]	*N* = 539 women, 12–17 years old	USA	RCT	Same-day start of OCPs with DOT	Standard OCP start	Starting second pill pack	**OR** **=** **1.80 (95% CI: 1.10–3.30)**
							Self-reported OCP continuation at 6 months	OR = 1.1 (95% CI: 0.70–1.80)
2007	Westhoff [25]	*N* = 1720 women, <25 years old	USA	RCT	Same-day start of OCPs with DOT	Standard OCP start	Starting second pill pack	**OR** **=** **1.5 (95% CI: 1.00–2.10)**
							Self-reported OCP continuation at 6 months	OR = 1.1 (95% CI: 0.80–1.30)
Social marketing campaigns								
2000	Van Rossem [94]	*N* = 868 women, 12–22 years old	Cameroon	QS	Social marketing and mass media campaign, youth clubs	SOC OCP provision	Self-reported OCP use preintervention and postintervention	15.0% Increase in OCP use in intervention setting versus 9.4% increase in control setting; χ^2^ = 0.96, *P* = 0.33

95% CI, 95% confidence interval; CRCT, cluster randomized controlled trial; DOT, directly observed therapy; OCP, oral contraceptive pills; OR, odds ratio; QS, quasi-experimental; RCT, randomized controlled trial; RR, relative risk; SOC, standard of care.

a Bold text denotes a statistically significant intervention effect.

Participants in one randomized controlled trial (RCT) who received daily text messages about OCP use for 6 months and two-way text message communication with providers to address their questions [22] had 44% greater odds (OR: 1.4; 95% CI: 1.0–2.0) of OCP continuation than those in the control.

In a study of multi-month pill dispensing with 85 adolescent girls and young women, participants in the intervention arm who received seven pill packs had seven-fold higher odds of OCP continuation (OR: 7.6; 95% CI: 2.4–24.0) than those receiving three pill packs after 6 months [23].

Two randomized trials examined the influence of same-day start of OCPs on continuation [24,25]. In these studies, intervention participants took their first dose of OCP while in the clinic regardless of menstrual cycle. Control participants were given pill packs and advised to start OCPs at the onset of their next menstrual cycle. Although neither trial found statistically significant effects after 6 months, both showed significant effects of same-day OCP start on self-reported completion of one OCP pack and continuation to a second pill pack by 3 months (OR: 1.8; 95% CI: 1.1–3.3 [24]; OR: 1.5; 95% CI: 1.0–2.1 [25]), suggesting that same-day starts may have a short-term effect. Both trials had 15–25% loss to follow-up by the 6-month interview and only 25–40% of participants reported OCP use after 6 months.

Three RCTs of counseling interventions showed contrasting results. Two showed improved OCP adherence through either clinic [27], or school-based counseling with decentralized OCP dispensing [26]. However, the school-based intervention did not impact OCP use among younger students (9th graders), potentially because the analysis was restricted to sexually active students who were less prevalent in this younger group [26]. Conversely, a third school-based cluster RCT with 7th graders found a significant negative effect of counseling whereby participants in the intervention arm had lower OCP use compared with the control (OR: 0.57; 95% CI: 0.36–0.91) [28]. The authors hypothesized that their findings could be explained by their choice of pregnant teens as the peer counselors, who may have had an unintended effect on OCP use [28]. The enhanced counseling interventions in these trials included one-time clinic-based or school-based sessions on OCP dosing, side effects, barriers and facilitators to OCP use, and contraceptive goal setting during the OCP initiation visit.

Several studies incorporated mHealth approaches to support enhanced counseling which did not demonstrate an effect on OCP use. These included one RCT of regular counseling phone calls [29] and another testing a menu-driven computer program for one-time tailored patient counseling [30].

### Summary of key limitations from oral contraceptive pill use interventions

Many studies in this review had limitations which are summarized here given their potential impact on the conclusions of this review. Six studies had loss to follow-up rates between 20 and 50%, which hampered the measurement of the intended outcome [23–25,27,31,32]. Two studies with enhanced peer-based counseling reported differential loss to follow-up and/or a lack of exchangeability between arms (the groups receiving enhanced counseling differed from the control in baseline characteristics which may also influence intervention engagement and OCP use) making the interpretations of the effects of peer-based counseling difficult [32,33]. Neither found a statistically significant effect [32,33]. With the exception of two studies that used pill counts and medical records [29] and urine samples [32] to assess OCP use, all studies relied on self-reported OCP use as their primary outcome, which could bias intervention effects if control participants felt less comfortable disclosing true OCP use compared with intervention participants. Six studies reported data on unintended pregnancies as a secondary outcome but the numbers of pregnancies were too small to compare between groups [24,25,29,30,34]. Finally, three studies did not disaggregate between female participants who were asked directly about their own OCP use and male participants who were asked about partners’ OCP use [28,33,34], which affects their interpretability.

### Effects of interventions on youth antiretroviral therapy, diabetes and asthma medication adherence

Across several systematic reviews, enhanced counseling (whether in groups, families, or computer-delivered), and phone-based support (e.g. one-way and two-way text messages), improved ART adherence [35–42], asthma symptom control [43–46], and HbA1c levels (a measure of better glycemic control) among people with diabetes [43,50–53]. Peer support interventions showed significant effects for ART [36,42], but neither peer-based nor school-based interventions were efficacious for asthma symptom control [44,47–49]. Adolescent-friendly services [36] were also effective for ART adherence, as was telemedicine for providers for diabetes [52,53]. Detailed results for each health condition are provided in Table 3 and Supplementary Appendix 1.

**Table 3:** Summary of systematic reviews focused on interventions to improve antiretroviral therapy, asthma, and diabetes medication adherence among adolescents and young adults (*N* = 26).^a^

Year	Author	Location^b^	*N* studies^b^	Intervention(s)^b^	Control(s)^b^	Primary outcome^b^	Key findings^b^
ART medication adherence							
2018	Ridgeway [35]	South Africa, Thailand	2	Enhanced group and family-based counseling	SOC counseling	Self-reported ART adherence; pill counts; pharmacy refills at 2–8 weeks post interventions	**Group counseling intervention:** χ^**2**^ **=** **14.7, df** **=** **1, *P* value** **<** **0.001Family-based counseling intervention: *P* value** **=** **0.05**
2018	Henny [37]	USA	1	Computer-delivered counseling	Active nutrition and physical activity control	Self-reported 7-day and weekend ART adherence at 6 months	**7-Day outcome: Cohen's *d*** **=** **0.49, *P*** **<** **0.05Weekend outcome: Cohen's *d* =** **0.66, *P*** **<** **0.01**
2017	Navarra [38]	USA	2	Phone-based counseling; web-based counseling	SOC counseling	Self-reported 7-day, weekend, and 30-day ART adherence at 6–12 months	**7-Day outcome: Cohen's *d* =** **0.49, *P*** **<** **0.05Weekend outcome: Cohen's *d* =** **0.66, *P*** **<** **0.0130-Day outcome: *P*** **=** **0.007**^b^
2017	Schaefer [43]	USA	1	Enhanced counseling	SOC counseling	HIV viral load at 6 and 9 months	**6 Months: *P*** **=** **0.03**9 Months: *P* > 0.05
2016	Shaw [40]	USA	1	Phone-based counseling	SOC counseling	30-Day ART adherence at 6–12 months	***P*** **=** **0.007**^c^
2015	Claborn [39]	USA	1	Computer-delivered counseling	Active nutrition and physical activity control	Self-reported 7-day and weekend ART adherence at 6 months	**7-Day outcome: Cohen's *d* =** **0.49, *P*** **<** **0.05Weekend outcome: Cohen's *d* =** **0.66, *P*** **<** **0.01**
2015	MacPherson [36]	USA, South Africa, France, Kenya, Mozambique, Tanzania, Rwanda	4	Adolescent-friendly clinic; longer clinic hours; family-based therapy; peer support	SOC ART provision, clinic services, and counseling	Gaps in ART coverage in last 12 months and cumulative incidence of ART initiation; missed last dose in past 3 months; proportion with undetectable viral load at 24 months	**Adolescent friendly-clinic intervention and gaps in coverage outcome: RR** **=** **5.56 (95% CI: 1.20–25.0)**Longer clinic longer hours intervention and ART initiation: HR = 1.06 (95% CI: 0.89–1.27)Family-based therapy intervention and missed last dose outcome: *P* = 0.05Peer support intervention and undetectable viral load outcome: *P* = 0.06^c^
2013	Arrivillaga [41]	USA	1	Phone-based counseling	SOC counseling	30-Day ART adherence at 6–12 months	***P*** **=** **0.007**^c^
2011	Bain-Brickley [42]	France	1	Peer support	SOC counseling	Undetectable viral load at 24 months	*P* = 0.06^c^
Asthma medication adherence							
2019	Ramsey [45]	USA	1	Web-based counseling	Generic asthma websites	Symptom days, symptom nights, hospitalizations at 12 months	**Symptom days: aRR** **=** **0.50 (95% CI: 0.40–0.80); symptom nights: aRR** **=** **0.40 (95% CI: 0.20–0.80); hospitalizations: aRR** **=** **0.20 (95% CI: 0.20–0.90)**
2018	Lancaster [46]	USA	1	Web-based counseling	SOC counseling	Symptom days at 12 months	**aOR** **=** **0.49 (95% CI: 0.24–0.79)**
2018	Ng [49]	USA	1	School-based care	SOC counseling	Symptom days at 12 months	aRR = 0.88 (95% CI: 0.74–1.04)
2017	Kew [47]	USA	1	Peer support	SOC counseling	Self-reported adherence, electronically measured adherence at 10 weeks	*P* = 0.929^c^
2017	Schaefer [43]	USA	1	Enhanced counseling with phone support	SOC counseling	Asthma symptoms at 3 months	**Difference: 10.94 (95% CI: 1.63–20.25); Cohen's *d* =** **0.96**
2016	Mosnaim [44]	USA; United Kingdom	4	School-based care; web-based counseling; MST; enhanced counseling	SOC counseling	Asthma symptom control and symptom days at 12 months; phone diary medication adherence at 7 months; self-reported adherence at 8 weeks	School-based care intervention and asthma control outcome: RR = 0.97 (95% CI: 0.91–1.04)**Web counseling intervention and symptom days outcome: RR** **=** **0.80 (95% CI: 0.60–1.00)MST intervention and adherence outcome: β** **=** **0.18 (95% CI: 0.02–0.34)**Enhanced counseling intervention and mean adherence outcome: 4.4 versus 4.3, ratio of 1.02
2011	Salema [48]	The Netherlands; United Kingdom	2	Enhanced counseling; school-based care	SOC counseling	Self-reported adherence and asthma symptom control	Enhanced counseling intervention and self-reported adherence outcome: 7.7 intervention versus 6.7 control; ratio of 1.15, *P* = 0.05School-based care intervention and asthma control outcome: RR = 0.97 (95% CI: 0.91–1.04)
Diabetes medication adherence							
2019	Virella Pérez [55]	Canada, Denmark	2	Phone and tablet-based support	SOC counseling	Percent changes in HbA1c levels at 12 months	All *P* > 0.05
2018	Ng [49]	United States	2	Enhanced counseling; web-based counseling	SOC counseling	Percent change in HbA1c levels between 6 and 12 months	Enhanced counseling: *P* > 0.05Web-based counseling: *P* > 0.05
2018	Feldman [50]	USA, Denmark, United Kingdom	11	Enhanced counseling (*N* = 9); MST (*N* = 2)	SOC counseling; waitlist control; nutritional counseling	Mean change in HbA1c levels between 3 and 12 months	**Enhanced counseling intervention: Cohen's *d* ranged from −1.57 to 0.38, all *P*** **<** **0.05MST intervention: Cohen's *d* ranged from 0.24 to 0.64, all *P*** **<** **0.05**
2017	Charalampopoulos [51]	United Kingdom	5	Phone support (*N* = 1); enhanced counseling	SOC counseling	Mean change in HbA1c levels between 5 days and 12 months	Pooled risk difference from meta-analysis with five adolescent-focused studies: −0.06 (−0.21 to 0.09); relative risk estimates ranged from −0.38 to 0.58 and one *P* < 0.05
2017	O’Hara [58]	Italy, Germany, United States	4	Transition coordinators; adolescent friendly clinic; enhanced education; computer management system	SOC counseling and medication services	Percent change in HbA1c levels between 3 and 12 months	Transition coordinator intervention: mean difference of 0.01, *P* > 0.05Adolescent friendly clinic intervention: *P* > 0.05^c^Enhanced education intervention: mean difference of −0.64% (95% CI: −0.79 to −0.50%)Computer management intervention: *P* > 0.05^c^
2017	Schaefer [43]	United Kingdom, United States	3	Enhanced counseling	SOC counseling	Mean change in HbA1c levels between 3 and 12 months	***P*** **<** **0.05**^c^
2017	Schultz [59]	Italy, Australia, Spain, Denmark, United Kingdom, United States, Israel, Canada	18	Transition coordinators	SOC counseling and medication services	Mean change in HbA1c levels between 3 and 12 months	Pooled risk difference from meta-analysis with 18 adolescent-focused studies: −0.11 (−0.31 to 0.08)
2016	Viana [52]	United States, Israel, Canada	6	Enhanced counseling, telecare, or educational intervention	SOC counseling	Mean change in HbA1c levels between 3 and 12 months	**Enhanced counseling intervention: mean difference** **=** **0.31 (95% CI: −0.60 to −0.02)**Telecare intervention: mean difference = −0.12 (95% CI: −0.27 to 0.02)Educational intervention: mean difference = −0.01 (95% CI: −0.20 to 0.20)
2015	Deacon [54]	United States	1	Text message adherence support	SOC counseling	Mean change in HbA1c levels at 1 month	Mean change of −0.06 in control versus −0.20 in intervention, *P* > 0.05
2014	Edwards [60]	United States	1	School-based care	SOC services	Mean change in HbA1c levels at 3 months	Mean change of 11.5 (95% CI: 9.3–14.0) in control versus 9.2 (95% CI: 7.4–11.0) in intervention
2013	Herbert [53]	United States, Norway, Austria	3	Phone and text message adherence support	SOC counseling	Mean change in HbA1c levels at 3–6 months	Mobile app intervention: *P* > 0.05^b^**Web-based app: 8.8% in intervention (no change in HbA1c levels from baseline) versus 9.9% in control (elevated HbA1c levels from baseline) (*P* for group and time interaction** **=** **0.006)Telemedicine support: HbA1c improvement from 9.0% at enrollment to 9.2% at month 6 for the telemedicine period versus continued HbA1c levels of 8.9% at enrollment and at month 6 for the conventional support period, *P*** **<** **0.05**
2011	Salema [48]	United States, Canada	4	Enhanced counseling	SOC counseling	Mean change in HbA1c levels at 3–12 months	Cohen's *d* estimates ranged from −0.20 to 0.48, all *P* > 0.05
2010	Hood [56]	United States, United Kingdom, India	9	Enhanced counseling	SOC counseling	Mean change in HbA1c levels at 3–12 months	Cohen's *d* estimates ranged from −0.55 to 0.59; pooled mean difference from meta-analysis with 9 adolescent-focused studies: 0.11 (95% CI: −0.01 to 0.23); all *P* > 0.05
2000	Hampson [57]	United States	7	Enhanced counseling	SOC	Mean change in HbA1c levels at 3–6 months	Cohen's *d* estimates ranged from −0.48 to 2.03; pooled mean difference from meta-analysis with 7 adolescent-focused studies: 0.33 (SD = 0.67)

95% CI, 95% confidence interval; ART, antiretroviral therapy; MST, multisystemic therapy; RR, relative risk; SOC, standard of care.

a The table has 30 rows but 26 studies, because Schaefer *et al.* is listed three times, Salema *et al.* is listed twice, and Ng *et al.* is listed twice.

b Data are shown only for the studies reported in the systematic reviews that included a comparison group (e.g. randomized controlled trials, quasiexperimental studies) and those that were conducted with 10–24 years old. Bold text indicates a significant effect. Measures of effect differ based on what was reported in the systematic review and/or included primary study (e.g. Cohen's *d*, relative risk, hazard ratio, *F* statistic). Pooled effect sizes from meta-analyses are reported and noted for those systematic reviews that included a meta-analysis restricted to ART, asthma, and diabetes intervention studies with 10–24 years old.

c Parent study only reported *P* value without a corresponding measure of effect.

## Discussion

The current article provides the first synthesis of adherence support interventions for daily medication use across a range of health conditions among adolescents and young adults, with the goal of informing future PrEP delivery for this priority population. Overall, interventions which resulted in improved adherence included text message reminders and two-way texting communication with providers, enhanced counseling, same-day pill starts and multi-month dispensing, peer support, and youth-friendly clinics. There was no evidence of effectiveness for financial incentives, directly observed therapy, broad social marketing campaigns, or transition care coordinators (Table 4).

**Table 4:** Summary of key takeaways from this systematic review of adherence support interventions.

• Interventions which resulted in improved adherence included: text message reminders and two-way text message communication with providers, enhanced counseling, same-day pill starts and multi-month dispensing, peer support, and youth-friendly clinics
• mHealth and enhanced counseling interventions were effective across OCP, ART, diabetes, and asthma medication use. Evidence of effectiveness was more mixed for same-day pill starts, multi-month dispensing, peer support, and youth-friendly clinics, where intervention effectiveness varied by medication type
• We did not find evidence of effectiveness for financial incentives, directly observed therapy, broad educational or social marketing campaigns, or transition care coordinators
• OCP use interventions may be the closest corollary to PrEP adherence given their focus on improving daily medication use and preventing a health condition in an otherwise healthy population. Investigators may want to weight evidence from OCP studies more strongly than ART, diabetes, and asthma interventions included in this review. However, the effective interventions we identified across all four types of medication use have the potential to target commonly reported barriers to PrEP adherence for adolescent populations and hypothesized mediators of medication use based on the information–motivation–behavioral skills model, including low social support and stigma, low risk perceptions, difficulties with dosing regimens, side effects, and logistical challenges of visiting the clinic and taking PrEP in daily life
• Most included studies were conducted in the United States and Europe, and PrEP delivery programs in resource-limited settings should consider these findings in light of their context and target population needs

ART, antiretroviral therapy; OCP, oral contraceptive pill; PrEP, pre-exposure prophylaxis.

OCP use is perhaps the closest corollary to PrEP use because it is also a daily medication used for prevention of a health condition in an otherwise healthy population and is only necessary while a woman perceives a need. In addition, young women using OCPs and/or PrEP often report stigma related to their sexuality and perceived promiscuity as barriers to both medications [61–63]. OCP interventions generally targeted mediators of daily pill use (e.g., information, motivations, support, availability) that have been described as barriers to PrEP adherence and continuation among adolescents [5–7,15,16]. One OCP study found that daily one-way and two-way text messaging with providers significantly increased OCP continuation [22], and similar text-based interventions for HIV testing, ART use, and sexual and reproductive health services found that two-way text messaging with a provider improved adherence by increasing knowledge about a medication, motivation, and perceived support [64–68]. Two pilot studies and one PrEP demonstration project have already incorporated text message reminders and two-way text message provider communication in PrEP delivery in Kenya and Brazil and found that messages were an acceptable way for participants to report on PrEP adherence and communicate issues with their provider [12,69,70]. In addition, a PrEP adherence intervention in South Africa and Zimbabwe found that one-way and two-way text messages were feasible for scalable delivery to check in with participants and triage concerns for providers [71], showing promise for future widespread use of text reminders and two-way support services in PrEP programs with youth.

Multi-month OCP prescriptions significantly improved OCP continuation in one small study [23]. However, this approach may not work for PrEP adherence among adolescents given data from prior trials showing consistent drop-offs in PrEP adherence and continuation when clinic visits were switched from monthly to quarterly [5,6,8,9]. Adolescents and young adults have reported concerns about not having a place to discreetly store a large number of PrEP pill bottles and find it reassuring to check in with providers and peers at the clinic [7,72,73]. Moreover, guidelines for PrEP delivery stipulate that individuals should come back to the clinic every 3 months for HIV testing and do not advise providing more than 3 months of PrEP at a time [74]. Rather than significantly extending the timing between pill pick-ups, PrEP programs could consider implementing decentralized provision (e.g. PrEP in schools or mobile sites). Telemedicine was effective at improving diabetes medication management [53] and may be another approach to improve PrEP delivery in contexts with busy clinics or in rural or remote regions where participants may have difficulty getting to a clinic. This is particularly salient in the context of restrictions due to COVID-19, where PrEP programs have had to rapidly adapt to telemedicine to continue services [75]. These approaches could potentially improve PrEP adherence for adolescents and young adults by reducing PrEP stigma from providers and the burden of clinic visits [76].

In at least one study for each health condition, enhanced counseling (e.g. motivational interviewing [77], problem-solving therapy [78,79], multisystemic therapy [80–82]) was effective at improving adherence. The counseling interventions varied in session number (e.g., one-time, 8–12 weekly sessions), length, and facilitator (e.g. lay counselor, peer). Family-based counseling significantly improved ART, diabetes, and asthma medication management, although it was not tested as an OCP intervention [35,44,50]. Family support is likely an important facilitator of PrEP use among young people who are still living with and financially dependent on parents or guardians [83–85], and may be feasibly integrated into PrEP delivery for youth who have strong relationships with family members and are willing to disclose their PrEP use. Interventions that did not show a significant effect on medication adherence were generally those that had fewer counseling sessions and/or measured the outcome further from the last counseling session.

We observed mixed findings on the impact of peer counseling; peer interventions were effective for ART adherence but not OCP, asthma, or diabetes medication use. Youth living with HIV likely face greater stigma than those using OCPs, asthma, or diabetes medication and it is possible that peer interventions for ART are efficacious by increasing support and motivations to adhere where HIV is highly stigmatized [86,87]. PrEP programs incorporating group counseling have already been shown to be acceptable among youth [71], and may lead to improved PrEP adherence particularly among those who experience stigma related to HIV and sexual behavior [63,88,89]. However, group counseling may not be appropriate for all youth, as some may not be able to attend group sessions or may be concerned about participating in a group where identity is based on a stigmatized behavior like sexual activity [71,90]. PrEP programs may consider hosting digital peer groups to reduce some of these barriers.

The current review has a number of limitations. We did not exclude studies for issues related to methodological rigor to be inclusive; however, a number of studies had limitations in outcome measurement (e.g. used self-reported adherence only), small sample sizes, and loss to follow-up. Excluding studies on the basis of quality such as those with imbalance across groups and high loss to follow-up, would have excluded interventions on peer-based counseling, enhanced counseling with motivational interviewing, and same-day OCP starts, but would not have substantially change our conclusions as we did not otherwise find strong evidence in support of peer-based counseling and motivational interviewing for OCP use, and same-day pill starts did not appear to have an impact on long-term OCP continuation. In addition, we base our conclusions about promising interventions on the magnitude of effect sizes and statistical significance. Despite these limitations, when taken together, the results provide useful indications of which interventions may be successful among youth. A majority of studies were conducted in the United States and Europe (particularly OCP, asthma, and diabetes studies), making it difficult to extrapolate results to resource-limited settings. While information from effective OCP, ART, diabetes, and asthma interventions may be useful to inform PrEP interventions for young people, there are key differences in populations, settings, provider competencies, stigma, and motivations that may influence whether these are successful with PrEP. Interventions for OCP, ART, diabetes, and asthma medication adherence largely target similar theoretical mediators of medication use, which have also been described as barriers to PrEP use, but some theoretical constructs of behavior change may be more relevant for PrEP adherence than these medications. For example, motivations to use PrEP may differ from motivations to use diabetes medication if consequences of nonadherence are seen to be more severe for one health condition versus another or normative beliefs about medication differ by condition. Moreover, ‘effective’ PrEP adherence is complex and PrEP needs may change as HIV risk and sexual behavior change. Interventions that improve ART, diabetes, and asthma medication management therefore may not be as appropriate for PrEP, compared with OCPs which also do not need to be taken with sustained, lifelong high adherence [91].

In conclusion, we identified a number of effective interventions to support daily medication adherence among youth that may be relevant for PrEP, including enhanced counseling, text messages and phone-based counseling, same-day pill starts and multi-month dispensing, peer support, and youth-friendly clinics. These interventions have the potential to target commonly reported barriers to PrEP adherence and mediators of PrEP use for adolescent populations [15,16,92]. However, the intervention choice will depend on context and population needs. Long-acting HIV prevention and coformulated contraceptives and HIV prevention products may become available in the next few years, which will provide method choice for young people but still requires adherence support. Similarly, the shorter, four-pill event-driven PrEP dosing regimen for young MSM requires only short-term adherence [95,96]. Future PrEP programs could focus on evaluating adherence support interventions outlined in this review, paying particular attention to those that target theoretical mediators of medication adherence and address empirically identified barriers to PrEP adherence, with the ultimate goal of developing effective interventions to achieve substantial reduction in new HIV infections among adolescents and young adults worldwide.

## Acknowledgements

We are grateful for the dedication of the thousands of young people who have participated in oral contraceptives, ART, diabetes, and asthma clinical trials and adherence studies around the world.

Author contributions: S.D. and R.B. conceived the study. S.D., R.B., B.K., L.-G.B., C.C., S.H., S.D.-M., and J.V. developed the systematic review protocol. J.V. conducted the systematic review with support from S.D. B.K., L.-G.B., C.C., S.H., S.D.-M., R.B., and S.D. provided oversight of the methods and contributed to the interpretation of results. J.V. prepared the first draft of the article and all authors edited, reviewed, and approved the final article. All authors have made substantial contributions to the work, drafted or revised it, and agree to be accountable for all aspects of the work.

Support for this work was provided by the WHO through Unitaid. J.V. was supported by the National Institute of Mental Health of the US National Institutes of Health (grant F31 MH113420) and the National Institute of Allergy and Infectious Diseases (grant T32 AI007140). The results and interpretation presented here do not necessarily reflect the views of the study funders. The funder of the study had no role in data collection, analysis, interpretation, or writing of the report. The corresponding author had full access to all the data in the study and had final responsibility for the decision to submit for publication.

### Conflicts of interest

The authors report no potential conflicts of interest.

## Supplementary Material

Supplemental Digital Content
