# Supplementary material for: Interventions to improve daily medication use among adolescents and young adults: what can we learn for youth pre-exposure prophylaxis services?
Source: AIDS. 2020 Dec 14;35(3):463–75. doi: 10.1097/QAD.0000000000002777 (PMC7855564; doi:10.1097/QAD.0000000000002777)
Supplement: Supplemental Digital Content [file aids-35-463-s001.docx]

**Supplementary Material 1**

***Effects of interventions on youth ART adherence***

ART reviews explored the impact of enhanced counseling, adolescent-friendly services, and decentralized medication refills, on self-reported adherence, pharmacy refills, pill counts, HIV viral load, and clinic attendance, with assessment between 2 weeks and 2 years after intervention delivery. Across several reviews, group and family-based counseling [35,36], computer-delivered counseling [37–39], phone-based support [38,40,41], and adolescent-friendly services within decentralized clinics [36] all showed significant improvement in ART adherence (Table 3). However, one review did not find that longer clinic hours to accommodate youth schedules had a significant effect on ART initiation (HR=1.06; 95% CI: 0.89, 1.27) [36]. All five systematic reviews with computer- or phone-based counseling support or SMS reminder messages (six studies total from the five reviews) found significant intervention effects on ART adherence at six months which persisted through twelve months [37–41]. In addition, two reviews with peer support interventions found significant effects on undetectable viral load by 24 months [36,42]. More transient intervention effects were observed in one study with enhanced counseling using motivational interviewing which found significant effects on HIV viral load at six months that did not persist through nine months [43]. However, this study had a small sample size and differential loss to follow-up between the motivational interviewing and control groups, which could have biased findings and limited power to detect a significant intervention effect by nine months [43].

***Effects on asthma medication adherence and symptom control***

Systematic reviews of asthma interventions focused on improving symptom control and asthma-related complications (e.g., hospitalizations,) over 8 weeks to 12 months. Enhanced counseling with motivational interviewing, problem-solving therapy, and multisystemic therapy improved asthma symptom control in three randomized trials from two included systematic reviews [43,44]. These counseling approaches were particularly efficacious when delivered in conjunction with phone support (difference in asthma symptoms at three months: 10.94, 95% CI: 1.63-20.25) and web-based counseling tools (RR for days with symptoms from three systematic reviews: 0.80, 95% CI: 0.60-1.00; 0.50, 95% CI: 0.40-0.80; 0.49, 95% CI: 0.24-0.79) [43–46]. One trial of multisystemic therapy with 170 participants found that participants in the therapy groups had higher adherence to their asthma medication at seven months than those in control arm (β=0.18, 95% CI: 0.02-0.34)[44]. Peer support [47] and school-based interventions [44,48,49] did not significantly improve asthma symptom control in five trials from four reviews.

***Diabetes medication adherence***

We included 14 reviews on diabetes interventions among youth, which focused on diabetes management and explored intervention effects on mean and percent changes in hemoglobin A1c levels (HbA1c) between intervention and control groups over 1 to 12 months. Changes in HbA1c levels are a function of more regular glucose monitoring and improved adherence to glycemic control measures, and it is difficult to disentangle intervention effects directly on medication adherence and chronic disease management. Interventions that significantly improved HbA1c levels included enhanced counseling (e.g., motivational interviewing, multisystemic therapy, problem-solving therapy) [43,50–52], phone-based support [51,53], and telemedicine support for providers [52,53]. While effects of enhanced counseling and phone-based support were largely significant and resulted in improved HbA1c levels in individual studies, several systematic reviews did not report a significant pooled effect of SMS- or application-based adherence support [54,55] or enhanced counseling [48,49,56,57] on HbA1c levels. However, the interventions included in these meta-analyses generally had smaller sample sizes, differential loss to follow-up, substantial between-study heterogeneity, and shorter follow-up periods of only approximately one to three months compared with the more recent reviews which found significant effects across several studies of enhanced counseling and SMS message support. None of the systematic reviews examining the impact of interventions with transition coordinators from pediatric to adult care [58,59] and school-based counseling [60] demonstrated statistically significant pooled effects from individual studies included in the review for these intervention approaches.
